# The Type I NADH Dehydrogenase of *Mycobacterium tuberculosis* Counters Phagosomal NOX2 Activity to Inhibit TNF-α-Mediated Host Cell Apoptosis

**DOI:** 10.1371/journal.ppat.1000864

**Published:** 2010-04-22

**Authors:** Jessica L. Miller, Kamalakannan Velmurugan, Mark J. Cowan, Volker Briken

**Affiliations:** 1 Department of Cell Biology and Molecular Genetics, University of Maryland, College Park, Maryland, United States of America; 2 Maryland Pathogen Research Institute, University of Maryland, College Park, Maryland, United States of America; 3 Division of Pulmonary and Critical Care Medicine, Department of Medicine, University of Maryland, Baltimore, Maryland, United States of America; University of New Mexico, United States of America

## Abstract

The capacity of infected cells to undergo apoptosis upon insult with a pathogen is an ancient innate immune defense mechanism. Consequently, the ability of persisting, intracellular pathogens such as the human pathogen *Mycobacterium tuberculosis* (Mtb) to inhibit infection-induced apoptosis of macrophages is important for virulence. The *nuoG* gene of Mtb, which encodes the NuoG subunit of the type I NADH dehydrogenase, NDH-1, is important in Mtb-mediated inhibition of host macrophage apoptosis, but the molecular mechanism of this host pathogen interaction remains elusive. Here we show that the apoptogenic phenotype of MtbΔ*nuoG* was significantly reduced in human macrophages treated with caspase-3 and -8 inhibitors, TNF-α-neutralizing antibodies, and also after infection of murine *TNF^−/−^* macrophages. Interestingly, incubation of macrophages with inhibitors of reactive oxygen species (ROS) reduced not only the apoptosis induced by the *nuoG* mutant, but also its capacity to increase macrophage TNF-α secretion. The MtbΔ*nuoG* phagosomes showed increased ROS levels compared to Mtb phagosomes in primary murine and human alveolar macrophages. The increase in MtbΔ*nuoG* induced ROS and apoptosis was abolished in NOX-2 deficient (*gp91^−/−^*) macrophages. These results suggest that Mtb, via a NuoG-dependent mechanism, can neutralize NOX2-derived ROS in order to inhibit TNF-α-mediated host cell apoptosis. Consistently, an Mtb mutant deficient in secreted catalase induced increases in phagosomal ROS and host cell apoptosis, both of which were dependent upon macrophage NOX-2 activity. In conclusion, these results serendipitously reveal a novel connection between NOX2 activity, phagosomal ROS, and TNF-α signaling during infection-induced apoptosis in macrophages. Furthermore, our study reveals a novel function of NOX2 activity in innate immunity beyond the initial respiratory burst, which is the sensing of persistent intracellular pathogens and subsequent induction of host cell apoptosis as a second line of defense.

## Author Summary

*Mycobacterium tuberculosis*, the causative agent of tuberculosis, is highly adapted to survive in macrophages of its human host. Host cell suicide is an ancient host cell defense mechanism employed by organisms to wall off invading pathogens. *M. tuberculosis* manipulates infected cells to inhibit host cell death but the molecular mechanism of this interaction has not been elucidated. Here we describe that *M. tuberculosis* uses an enzyme complex (NDH-1) usually needed for energy generation in order to neutralize the NOX-2 enzyme-mediated production of toxic oxygen radicals (ROS) by the host cell. We demonstrate that an *M. tuberculosis* mutant deficient in NDH-1 accumulates ROS inside the macrophage which induces the secretion of an inflammatory cytokine (TNF-α) and subsequent host cell death. The increase of ROS is dependent upon functional NOX-2, since host cells missing a NOX-2 component do not undergo cell death upon infection with the mutant. We propose that a novel function of the host cell NOX-2 complex is to allow sensing of intracellular pathogens by the host cell in order to commit suicide and thus limit bacterial survival.

## Introduction

The phagocytic NADPH-oxidase (NOX2-complex or phox) resides on phagosomes and has been shown to be involved in microcidal activity in phagocytes. NOX2 is the original member of the NOX family of reactive oxygen species (ROS)-generating NADPH oxidases, which now includes NOX1-NOX5, DUOX1 and DUOX2 [1], [2]. The multicomponent NOX2 complex consists of two transmembrane proteins, gp91^phox^ and gp22^ phox^, and three cytosolic components, p40^ phox^, p47^ phox^ and p67^ phox^ [1], [2]. Additionally, the cytosolic GTPase Rac has to be recruited in order to form a fully active NOX2 complex [1]. The gp91^phox^ subunit, which is constitutively associated with gp22^ phox^, is a transmembrane redox chain that generates phagosomal superoxide by transferring electrons from cytosolic NADPH to phagosomal oxygen [1]. NOX2-generated superoxide can then be converted into a multitude of microcidal oxidants, including hydrogen peroxide and hypochlorous acid, which are important components of the bactericidal activity of the macrophage phagosome [3]. However, NOX2 activity seems to serve a different function in phagosomes of dendritic cells, where it is important for efficient crosspresentation of antigens [4]. The significance of the NOX2-complex for innate immune response is illustrated by the development of chronic granulomatous disease (CGD) in human subjects that have genetic defects in components of the complex. CGD is characterized by greatly increased susceptibility to fungal and bacterial infections [5]. Correspondingly, mice deficient in the NOX2 subunits are much more susceptible to infections with bacterial pathogens such as *Salmonella typhimurium* for example [3], [5]. Not surprisingly, some pathogens have developed strategies to counter the NOX2 response by either inhibiting NOX2 assembly on the phagosome, as is the case for *S. typhimurium* [3] and *Helicobacter pylori* [6], or reducing steady-state levels of NOX2 components as illustrated by *Anaplasma phagocytophila* [7] or *Ehrlichia chaffeensis* [8] (for review [9]).

Programmed cell death (PCD), or apoptosis, plays an important role in the innate immune response (IR) against pathogens, a defense strategy that is evolutionarily conserved and extends even into the plant world[10]. Inhibition of host cell apoptosis has been extensively studied and there are numerous examples of viral proteins directly interfering with host cell apoptosis signaling[11]. Furthermore, an increasing number of protozoal pathogens have been shown to manipulate PCD signaling of infected host cells[12]. Finally, prokaryotic pathogens such as *Chlamydia*, *Legionella*, *Rickettsia*, and *Mycobacterium* among others have the capacity to inhibit host cell apoptosis signaling [13], [14].

*Mycobacterium tuberculosis* (Mtb) is an extremely successful human pathogen that manipulates host cells via multiple pathways in order to achieve survival[15], [16], [17]. The inhibition of host cell apoptosis by Mtb has been implicated as a potential virulence mechanism[18]. Indeed, an inverse correlation between the virulence of a mycobacterial species and their capacity to induce apoptosis of primary human alveolar macrophages was demonstrated[19]. Cells infected with virulent Mtb have also been shown to be more resistant to various apoptosis stimuli when compared to uninfected or avirulent strains of Mtb[18]. For example, Mtb-infected macrophages secrete soluble TNF-α-receptor in order to inhibit TNF-α-mediated host cell apoptosis induction [20]. Mtb-infection reduces the cell surface expression of Fas receptors, resulting in the resistance of the host cells to Fas-ligand induce cell death[21]. Infection with Mtb also induces the upregulation of the anti-apoptosis gene *mcl-1*, which confers resistance of cells to apoptosis induction via the host cell mitochondria[22]. Finally, it has recently been shown that Mtb can manipulate the surface of infected macrophages in order to favor a necrotic, rather than apoptotic, cell death outcome[23]. In macrophages infected with virulent MtbH37Rv, but not avirulent MtbH37Ra, the amino-terminal domain of annexin-1 is removed by proteolysis, preventing completion of the apoptotic envelop [24]. Similarly, cells infected with Mtb are less likely to induce host cell membrane repair, which is important for the induction of apoptosis and supports the induction of necrotic cells death and the subsequent dissemination of bacteria[24], [25].

While there is substantial evidence supporting the ability of Mtb to inhibit host cell apoptosis, a causal link between apoptosis inhibition and virulence of Mtb had not been established due to the lack of defined pro-apoptosis mutants. We have recently performed a “gain-of-function” genetic screen and identified three independent regions in the genome of Mtb that contain anti-apoptosis genes[26]. The deletion of one of the identified genes, the *nuoG* gene of Mtb, which encodes one of the 14 subunits of the type I NADH dehydrogenase (NDH-1), increased infection-induced apoptosis of macrophages and significantly reduced bacterial virulence in mice. These findings support a direct causal relationship between virulence of pathogenic mycobacteria and their ability to inhibit macrophage apoptosis[26]. Our findings are consistent with the identification of another anti-apoptosis gene (superoxide dismutase A) that plays an important role in the virulence of Mtb[27]. Finally, a third gene (Protein kinase E) with anti-apoptosis capacity has recently been described, but the impact of the deletion of this gene on bacterial virulence has not been established[28]. Altogether, the identification of multiple anti-apoptosis genes suggests that Mtb utilizes several strategies to inhibit the apoptotic response of the host cell; however the molecular mechanisms of these interactions have not been investigated.

The present study describes the investigation of the molecular mechanisms by which NuoG of Mtb inhibits host cell apoptosis. The use of TNF-α-neutralizing antibodies and specific caspase inhibitors on human macrophage cell lines, as well as the infection of bone-marrow derived macrophages (BMDM) of wild-type and *TNF^−/−^* mice demonstrated that NuoG is involved in inhibiting an extrinsic, TNF-α-dependent, apoptosis pathway. Furthermore, the pro-apoptotic phenotype of the *nuoG* mutant was abolished in the presence of both ROS scavengers and in the absence of a functional NOX2 system as demonstrated in BMDM and primary human alveolar macrophages. Altogether, our results reveal a novel function of the NOX2 system in helping the host macrophage in sensing persistent intracellular mycobacteria via increased phagosomal ROS levels and the subsequent induction of host cell apoptosis. This may constitute a second line of defense of the macrophage and it is intriguing to speculate that this NOX2 mediated apoptosis induction is equally important in the defense against other intracellular pathogens.

## Results

### The MtbΔ*nuoG* mutant induces apoptosis via an extrinsic, caspase-dependent pathway

**Figure 1:**
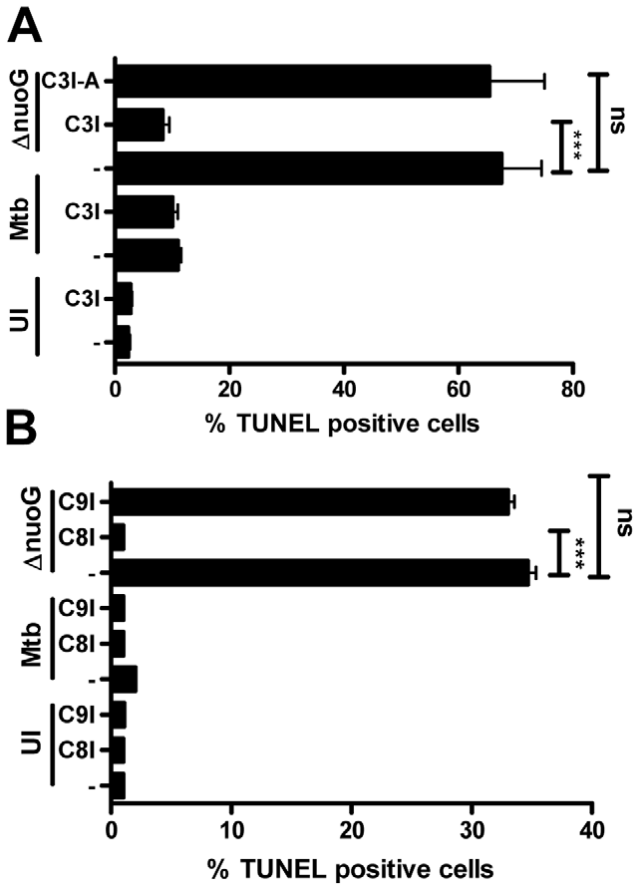
Mtb NuoG mediates inhibition of extrinsic but not intrinsic apoptosis pathways. (A and B) THP-1 cells were either infected with Mtb or the *nuoG* mutant (Δ*nuoG*) for 4 h at an MOI of 5, or left uninfected (UI). (A) Cultures were either treated with a 20 µM of Caspase-3 inhibitor (C3I), an inactive analog of the inhibitor (C3I-A) or medium only (−), and analysis of TUNEL+ cells by FACS was performed after 5 days. (B) As in (A), THP-1 cells were infected or left untreated (UT), and cultured with specific inhibitors of Caspase-9 (C9I), Caspase-8 (C8I) or in medium alone (−) for 3 days, followed by analysis of TUNEL^+^ cells. Statistical analysis was performed on three independent experiments (ANOVA with Tukey post-test) and significance is indicated as follows: *, 0.01<p<0.05; **, 0.001<p<0.01; ***, p<0.001.

We previously demonstrated that a Δ*nuoG* mutant of Mtb induced more apoptosis in host cells than wild type bacteria [26]. In order to analyze the mechanism of the NuoG/NDH-1 mediated host cell apoptosis inhibition, we first determined the involvement of caspases in the pro-apoptotic phenotype of MtbΔ*nuoG* using specific caspase inhibitors. PMA-differentiated THP-1 cells were pre-treated with Caspase-3 inhibitor (C3I) or a chemical analog with no inhibitor activity (C3I-A) at 20 µM for 3 h before infection, during infection, and after infection. Cells were either left uninfected, or were infected with Mtb or MtbΔ*nuoG*. After five days cells were harvested and stained for genomic DNA degradation using the fluorescent Terminal deoxynucleotidyl transferase dUTP Nick End Labeling (TUNEL) assay. The percentage of TUNEL positive cells was determined by flow cytometry analysis. This analysis revealed that the uninfected population contained low percentage of apoptotic cells (2.3±0.3%), Mtb infection slightly increased this amount to 11±0.6%. As expected from previously published results [26], cells infected with the *nuoG* mutant showed a very significant increase in apoptosis (67.5±7.0%). Interestingly, treatment of THP-1 cells with the C3I reduced the percentage of Δ*nuoG* induced apoptosis to 8.3±1.2%, whereas the C3I-A had no significant effect on apoptosis induction (65.3±9.7%)(Figure 1A). The C3I did not have an effect on the low level of Mtb-induced apoptosis, as 10.0±1.0% of C3I-treated Mtb-infected cells were TUNEL positive, suggesting that Mtb may be inducing low levels of apoptosis via a caspase independent mechanism. In order to determine if the *nuoG* mutant induces apoptosis via the extrinsic (i.e., death receptor mediated), or the intrinsic (i.e., mitochondrial) pathway [29], cells were treated with Caspase-8 and Caspase-9 inhibitors, respectively. The experimental conditions and analysis were identical to the previous experiment with the exception that cells were harvested 3 days post infection. Analysis of TUNEL staining after this shorter time period resulted in similar rates of apoptosis for Mtb infected and uninfected cells (2.1±0.1% and 1.1±0.1%, respectively). Treatment of these populations with either C8I or C9I had no effect. The *nuoG* mutant induced apoptosis in about 34.7±0.7% of cells, which was not significantly affected by the addition of C9I (33.2±0.6), but was significantly reduced by the addition of C8I to levels similar to uninfected cells (1.2±0.2%)(Figure 1B). These results indicated that the *nuoG* mutant induced host macrophage apoptosis via an extrinsic, caspase-8 dependent signaling pathway.

### Host macrophage TNF-α is important for the apoptogenic phenotype of the *nuoG* mutant

**Figure 2:**
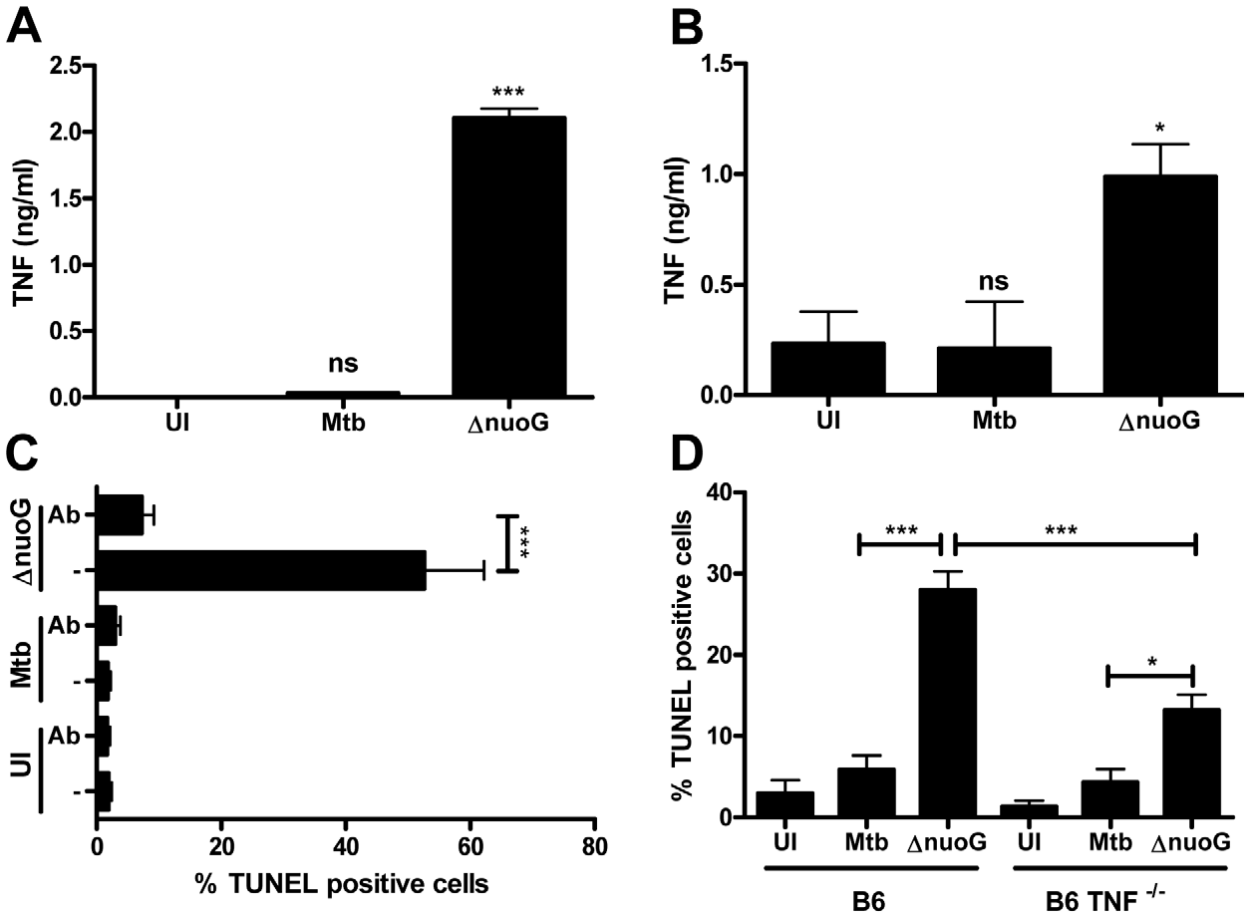
Mtb NuoG mediates inhibition of TNF-α-induced apoptosis and TNF-α secretion. (A and B) TNF-α secretion was measured in human THP-1 (A) or primary murine (B) macrophages 3 days post infection with Mtb and *nuoG* knockout bacteria by ELISA. Results in (A) are a representative example. (C) Macrophages were infected with wild type (Mtb) and *nuoG* knockout (Δ*nuoG*) bacteria in the presence of 5 µg of TNF-α-neutralizing antibodies. Apoptotic cells were quantified by TUNEL staining 5 days post infection. (D) BMDM derived from C57B/6 (B6) and *TNF-*α knockout mice (B6 *TNF^−/−^*) were infected with wild type and mutant bacteria and were assayed for apoptosis 5 days post infection.

TNF-α is of major importance for a successful host defense against mycobacterial infections, and has also been implicated in the apoptosis response to mycobacterial infection by the macrophage [30], [31]. Since TNF-α receptor signaling can result in cellular apoptosis, we tested whether autocrine TNF-α production and signaling were involved in apoptosis of MtbΔ*nuoG* infected THP-1 cells. We first determined if infection with MtbΔ*nuoG* resulted in an increase of secreted TNF-α. Supernatants from infected THP-1 (Figure 2A) and BMDM cells (Figure 2B) were collected 3 days post infection and levels of TNF-α were measured by ELISA. In both systems, MtbΔ*nuoG* infected cells secreted significantly more TNF-α than those infected with wild type (30 pg/ml to 2.1 ng/ml for Mtb and MtbΔ*nuoG* in THP-1 cells; 0.2 ng/ml to 1 ng/ml for Mtb and MtbΔ*nuoG* in murine cells). Having established the presence of higher levels of TNF-α, we next evaluated the effect of TNF-α signaling on the pro-apoptotic phenotype of MtbΔ*nuoG*. This was first addressed by addition of human TNF-α-specific, neutralizing antibodies (5 µg/ml) to the culture media of THP-1 cells during and after infection. The anti-TNF-α-antibody significantly inhibited macrophage apoptosis induced by MtbΔ*nuoG* infection, as the percentage of apoptotic cells was reduced from 62.3±9.6% to 7.2±1.96% after addition of antibody (Figure 2C). PCD in uninfected or Mtb infected cells was not significantly affected by the addition of antibodies (Figure 2C). The involvement of TNF-α in the pro-apoptotic phenotype of the *nuoG* mutant was further analyzed by utilizing BMDM from *TNF-*α*^−/−^* mice. The *nuoG* mutant induced apoptosis in 28±2.3% of wild type C57B/6 (B6) cells, as compared to 5.8±1.8% of Mtb infected cells (Figure 2D). In contrast, the pro-apoptotic phenotype of the *nuoG* mutant was partially complemented in *TNF^−/−^* BMDM, resulting in levels of apoptosis of 13.3±1.9%, where as Mtb infected cells were not significantly different at 4.3±1.6%. Overall, these experiments confirmed the involvement of TNF-α in the pro-apoptosis phenotype of the *nuoG* mutant.

### Host macrophage NOX2-derived reactive oxygen species are necessary for MtbΔ*nuoG*-induced apoptosis and increase in TNF-α secretion

**Figure 3:**
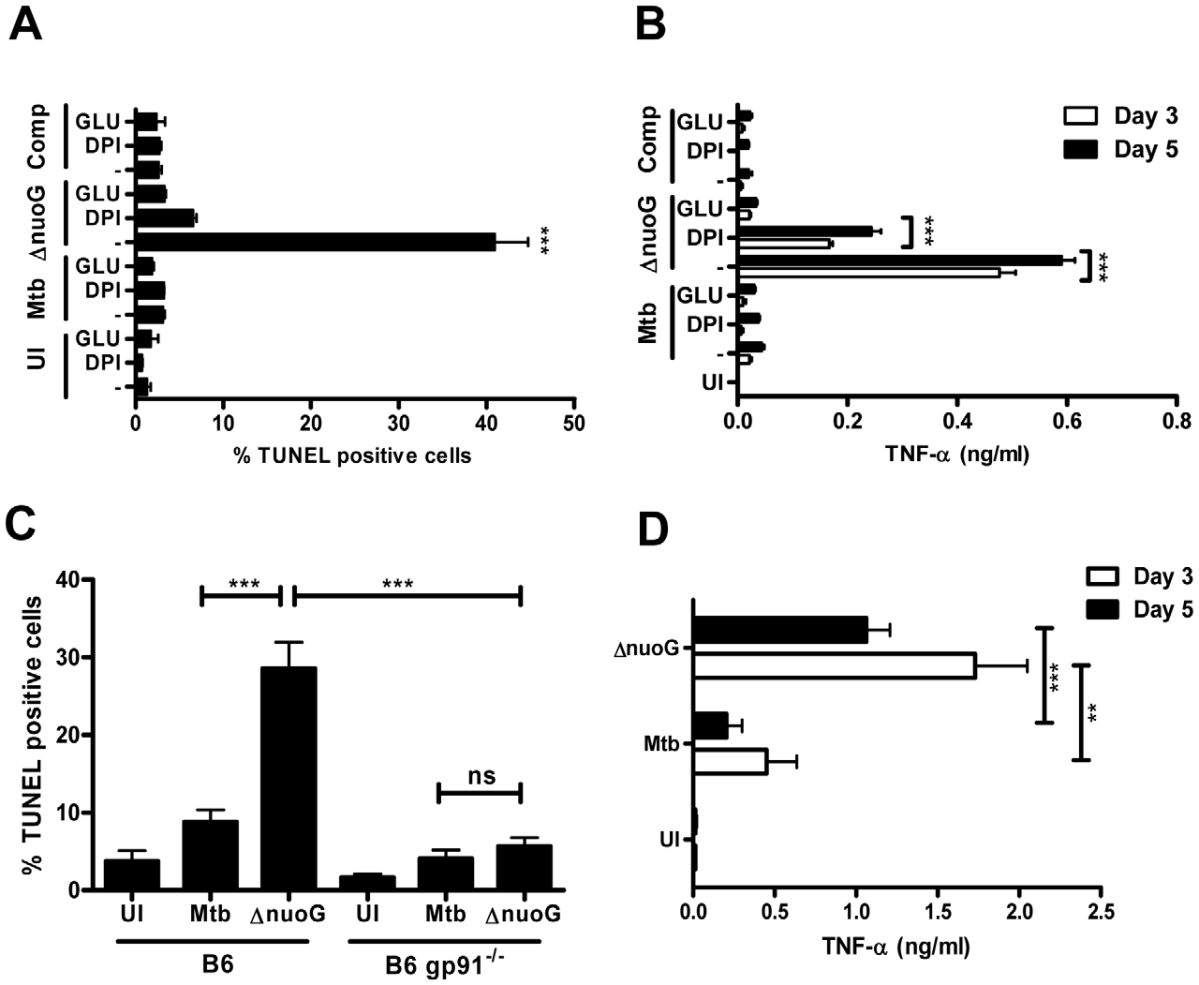
Mtb NuoG mediates inhibition of ROS-dependent induction of apoptosis. (A) THP-1 cells were infected with wild-type (Mtb), Δ*nuoG*, or complemented mutant (Comp) strains of Mtb or left uninfected (UI). Cultures were incubated in medium alone (−), or in medium containing 15 mM glutathione (GLU) or 10 µM DPI. Apoptotic cells were quantified via TUNEL staining 5 days post infection. (B) Supernatants of the cultures from (A) were harvested on indicated days, and levels of TNF-α were determined by ELISA. (C) Macrophages derived from wild type C57B/6 (B6) or NOX2 deficient *gp91* knockout mice (B6 *gp91* ^−/−^) were infected with Mtb or MtbΔ*nuoG*. Apoptosis was assayed after 5 days by TUNEL staining. (D) Supernatants of B6 *gp91* ^−/−^ infected macrophages from experiment shown in (C) were harvested after indicated days and levels of TNF-α were determined by ELISA.

Reactive oxygen species (ROS) are involved in shifting the balance of TNF-α-R1 mediated signaling from anti-apoptotic to pro-apoptotic [32], [33]. We investigated the role of ROS in MtbΔ*nuoG* induced apoptosis by utilizing a general ROS scavenger (the antioxidant glutathione) and an oxidase inhibitor (diphenylene iodonium or DPI) during infections of THP-1 cells [1]. THP-1 cells were incubated with 15 mM glutathione or 10 µM DPI 3 hours prior to and throughout infection with Mtb and MtbΔ*nuoG*. Untreated cells infected with the *nuoG* mutant induced apoptosis in about 40.95±3.8% in the population, as compared to 1.3±0.4% in uninfected, and 3.1±0.2% in Mtb infected cells (Figure 3A). The presence of DPI and glutathione reduced apoptosis induced by the mutant to 6.6±0.4% and 3.3±0.2% of cells, respectively (Figure 3A). Thus, both of these agents greatly suppressed apoptosis induced by MtbΔ*nuoG* in THP-1 cells, a finding consistent with a strong dependence of the apoptotic death response on ROS accumulation (Figure 3A) [33]. These inhibitors can also potentially affect cellular NO levels but we determined, using the Griess assay, that THP-1 cells produce no significant increase in NO after infection with the bacteria (Figure S2). Increased ROS levels in the cytosol can also lead to increased gene transcription of an array of genes involved in oxidative stress and immunity, including TNF-α [32]. For that reason, the TNF-α levels in the supernatant of infected THP-1 cells were analyzed after 3 and 5 days by ELISA. Insignificant amounts of TNF-α were detected in supernatants of uninfected cells, and only low concentrations of TNF-α (below 50 pg/ml) were detected in supernatants of cells infected with Mtb or the complemented *nuoG* mutants strains (Figure 3B). In contrast, the *nuoG* mutant increased secretion of TNF-α by a factor of 10 to 0.5–0.6 ng/ml. This increase was partially reduced to about 0.1–0.2 ng/ml by treatment of the cells with DPI, and almost completely reduced by the addition of glutathione (0.02–0.03 pg/ml)(Figure 3B). Thus, the increase of intracellular ROS induced by infection of cells with the *nuoG* mutant is required for the increase in TNF-α secretion by infected cells.

Next, we addressed the question of the subcellular origin of ROS during MtbΔ*nuoG* infection. The mitochondrial respiratory chain complex I is an important generator of cellular ROS that is shared by all cells types and might be at the origin of mitochondrial-induced host cell apoptosis. However, the NADPH oxidases are also potent inducers of cellular and extracellular ROS. In macrophages, the phagocyte NADPH oxidase, NOX2, is recruited to phagosomes and generates the production of superoxide in the lumen of the phagosome. These superoxides and their derivates are thought to be important for the killing of ingested bacteria, although their role in pathogenesis is not completely understood. In order to address the importance of NOX2 in the pro-apoptotic phenotype of the *nuoG* mutant, we utilized BMDM derived from mice deficient in NOX2 activity due to the deletion of the major transmembrane subunit of the NOX2 complex, gp91^phox^ (*gp91^−/−^*). The *nuoG* mutant induced significantly more apoptosis than Mtb in macrophages of wild type C57Bl/6 mice, 28.6±3.4% versus 8.8±1.6%, respectively (Figure 3C). Importantly, this increase was abolished when *gp91^−/−^* BMDM were used as host cells, since only 5.7±1.1% of MtbΔ*nuoG* infected cells were apoptotic compared to 4.1±1.1% of Mtb-infected cells. Therefore, the presence of functional NOX2 is required for the pro-apoptotic phenotype of the *nuoG* mutant of Mtb. Interestingly, the absence of NOX2 in infected primary macrophages did not result in the reduction of TNF-α secretion as nuoG infected cells secreted more TNF-α (day 3: 1.7±0.3 ng/ml; day 5: 1.1±0.2 ng/ml) than those infected with Mtb (day 3: 0.5±0.2 ng/ml; day 5: 0.2±0.1 ng/ml).

### Macrophage infection with MtbΔ*nuoG* induces phagosomal ROS accumulation

**Figure 4:**
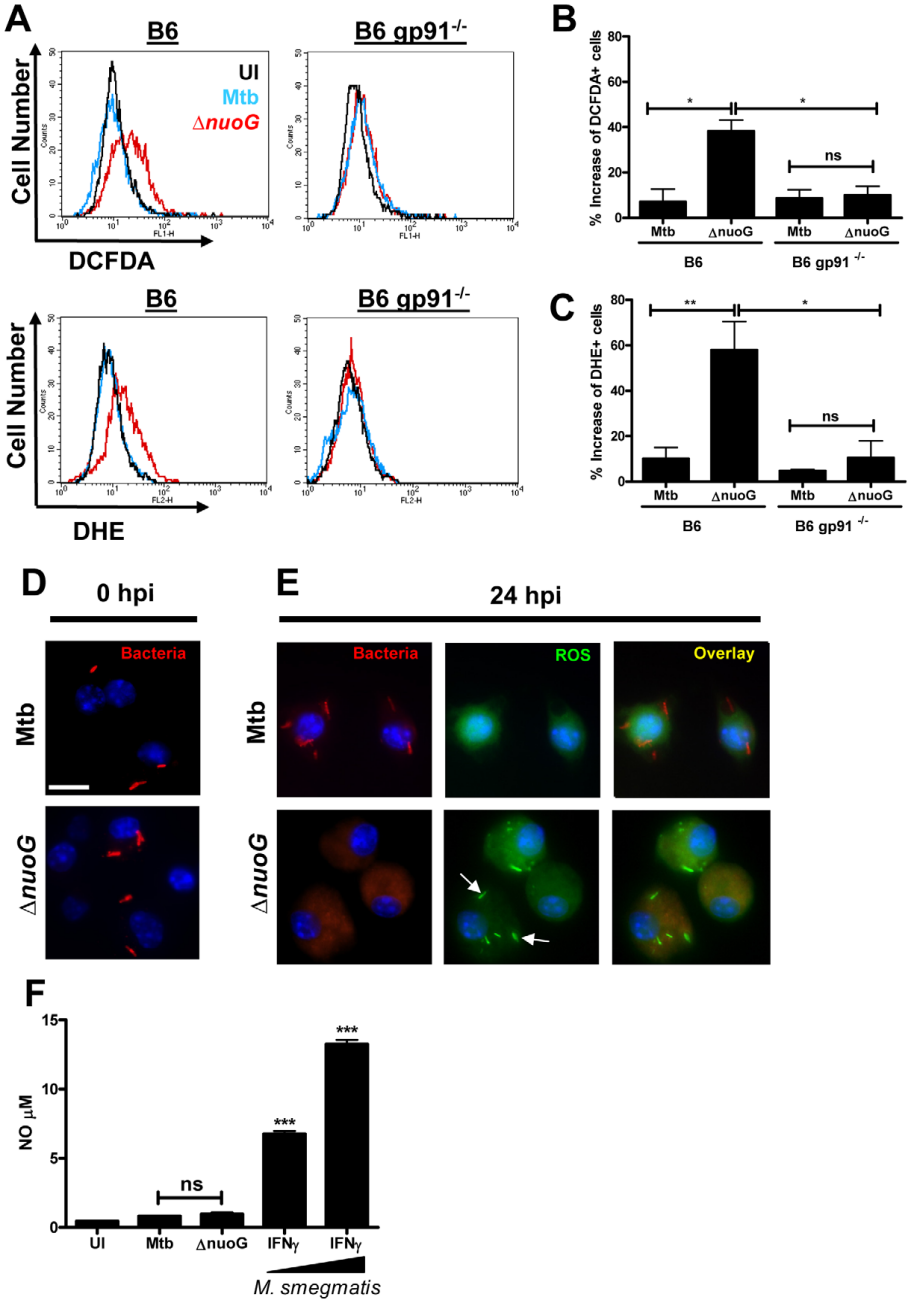
Mtb NuoG mediates inhibition of infection-induced phagosomal ROS production. (A) Macrophages from C57B/6 (B6) or gp91 deficient mice (B6 *gp91* ^−/−^) were infected with Mtb or Δ*nuoG* and stained 24 hours post infection (hpi) with the ROS sensitive dyes DCFDA and DHE, which are more sensitive for H_2_O_2_ and O_2_^-^ respectively. ROS production was measured by flow cytometry. (B and C) Quantification of the increase in ROS levels as detected by DCFDA (B) or DHE (C) fluorescence intensities. Net increases in individual fluorescence intensities were obtained by subtracting the fluorescence intensity distributions of untreated cells from the corresponding Mtb or Δ*nuoG* infected distribution. (B and C). C57/B6 macrophages were infected with wild type (Mtb) and NuoG deficient (Δ*nuoG*) bacteria labeled with the lipophilic dye DiI. Cells were visualized via fluorescence microscopy (scale bar  = 10 µm) at (D) 0 hpi, or stained with DCFDA and visualized at (E) 24 hpi. Arrows indicate the accumulation of ROS in the phagosome. Note that the DiI fluorescence is lost in the presence of ROS (E, lower row), but not in the absence of ROS (D and E, upper row). (F) Quantification of NO produced by infected macrophages. BMDMs from B6 mice were infected with Mtb or Δ*nuoG* and NO concentrations in the cell supernatant were determined by the Griess assay. Supernatants from macrophages primed with IFNγ and exposed to increasing concentrations of heat-killed *M. smegmatis* were used as positive controls. (A,D,E) Representative samples shown.

If the ROS responsible for the pro-apoptotic phenotype of the *nuoG* mutant originate from NOX2, then macrophages infected with MtbΔ*nuoG* should have higher intracellular levels of ROS than those infected with Mtb. In order to address this hypothesis, two dyes for detection of ROS were used: DCFDA, which is more sensitive to H_2_O_2_, and DHE, which is more sensitive to O_2_^-^. Macrophages were infected and after 24 h the amount of ROS was detected in uninfected, Mtb and MtbΔ*nuoG* infected cells using flow cytometry analysis. Mtb infection induced only slightly elevated levels ROS as detected either by DCFDA or DHE since the histogram overlays closely with that of uninfected cells (Figure 4A–4C). Conversely, both dyes detected a significant increase in ROS levels after infection of wild type cells with the *nuoG* mutant as depicted by the positive shift in fluorescence (Figure 4A–4C). The pro-apoptotic phenotype of the *nuoG* mutant was also observed under these conditions (Figure S1A). Importantly, this increase in ROS staining was abolished in *gp91^−/−^* BMDM, thus clearly indicating that ROS are being generated by the NOX2 complex (Figure 4A–4C). In order to directly observe ROS localization on a subcellular level, macrophages were infected with DiI-labeled mycobacteria (Figure 4D), stained with DCFDA, fixed, and analyzed by fluorescence microscopy. Only in the MtbΔ*nuoG* infected macrophages were phagosomes stained with the ROS sensor DCFDA, whereas phagosomes of Mtb-infected macrophages remained DCFDA negative (Figure 4E). This data also revealed that the DiI fluorescence is quenched in the presence of ROS and thus the bacterial staining is lost during infection with MtbΔ*nuoG*, but not during infection with Mtb (Figure 4E). Other dyes were used for external labeling of bacteria with similar results (Data not shown). These results not only confirmed the flow cytometry analysis in which an increase of ROS signal was detected only after infection of macrophages with the MtbΔ*nuoG* mutant (Figure 4A), but furthermore localized this increase of ROS to the host cell phagosome (Figure 4E). Nitric oxide (NO) can also oxidize DCFDA to induce fluorescence[34]. However, BMDMS infected with Δ*nuoG* did not produce significantly more NO (0.97±0.3 µM) than those infected with wild type Mtb (0.82±0.09 µM) and both values were only very marginally elevated compared to uninfected cells (0.45±0.04 µM). In contrast, IFNγ-activated macrophages infected with non-pathogenic *Mycobacterium smegmatis* induced very significant increases in NO levels (6.77±0.41 µM at MOI 3 and 13.25±0.30 µM at MOI 10)(Figure 4F). The overall NO production in the human THP-1 cells was low, even after IFNγ activation (Figure S2). Thus, the visualized increase of DCFDA fluorescence is likely due to oxidation by ROS.

### Primary human alveolar macrophages undergo apoptosis upon MtbΔ*nuoG* infection in a ROS-dependent fashion

**Figure 5:**
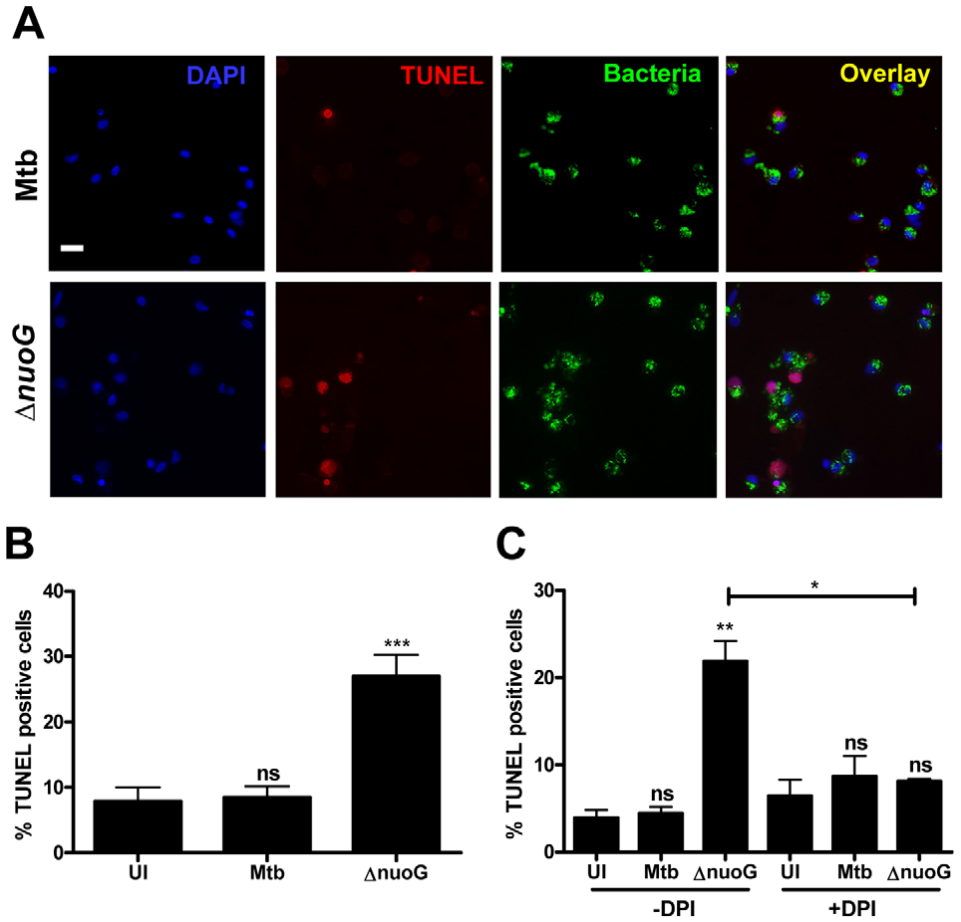
The pro-apoptotic phenotype of the *nuoG* mutant is conserved in primary human alveolar macrophages and is dependant on ROS. (A) Fluorescence microscopy of human alveolar macrophages infected with wild type (Mtb) or NuoG deficient bacteria (Δ*nuoG*) and stained with TUNEL 3 days post infection (scale bar  = 20 µm)(representative sample). (B) Quantification of TUNEL positive macrophages (500 cells counted per condition; average of 5 donors). (C) Human macrophages were infected in the presence of the NOX2 inhibitor DPI (10 µM) and assayed for apoptosis 3 days post infection (500 cells counted per condition; average of two donors).

**Figure 6:**
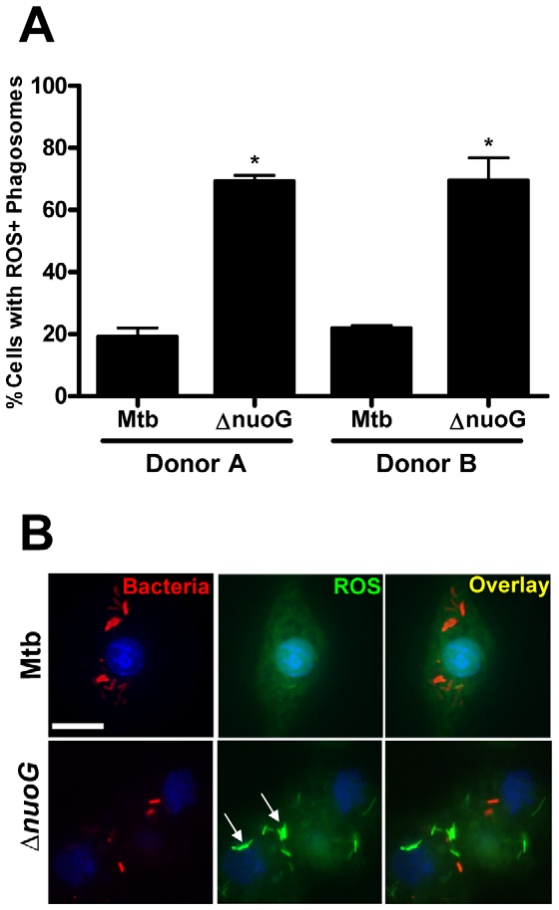
MtbΔ*nuoG* induces phagosomal ROS production in infected primary human alveolar macrophages. (A and B) Alveolar macrophages were infected with Mtb and *nuoG* knockout bacteria (Δ*nuoG*) and stained with the ROS sensitive dye DCFDA after 3 days. (A) Quantification is of cells containing one or more ROS positive phagosomes (two donors shown). (B) Fluorescence microscopy of DCFDA stained alveolar macrophages infected with DiI labeled bacteria (scale bar  = 10 µm)(representative sample). Arrows indicate phagosomal ROS accumulation.

In order to analyze if the ROS-dependent mechanism of apoptosis induction upon infection with the Δ*nuoG* is conserved in human cells, primary alveolar macrophages were used as host cells. Due to the source of the cells, only a limited number of cells were available, and therefore the apoptosis assay was adapted to be performed on slides which were analyzed by fluorescence microscopy. For each donor triplicate wells were infected with Mtb, MtbΔ*nuoG*, or were left uninfected. Cells were stained with TUNEL assay 3 days post infection (Figure 5A). Approximately 500 cells were counted on each slide in blinded fashion and the number of TUNEL positive cells was recorded (Figure 5B). Approximately 7.9±2.2% of uninfected macrophages were apoptotic, a percentage which was not significantly different from that of Mtb infected cells (8.5±1.7%). In contrast, there was roughly a 3fold increase in the percentage of apoptotic macrophages infected with MtbΔ*nuoG* (26.9±3.3%). These results were pooled from five different donors, indicating that the phenotype of NuoG-mediated apoptosis inhibition is consistently conserved among different human subjects. The dependence of this pro-apoptotic phenotype on the generation of intracellular ROS was analyzed in two different donors using the inhibitor DPI as described above. Approximately 5 times as many human cells infected with the *nuoG* mutant underwent apoptosis as compared to those infected with Mtb (21.9±2.4% and 4.5±0.8% respectively). However, this difference between the two strains was abolished by the treatment of cells with the inhibitor DPI, as about 8.7±2.3% of Mtb and 8.1±0.3% of Δ*nuoG* infected cells were apoptotic under these conditions (Figure 5C). These data strongly suggests that in primary human alveolar macrophages, as in murine BMDM, the NOX2 complex is critical for the pro-apoptotic phenotype of the *nuoG* mutant. Lastly, the intracellular ROS levels in Mtb or MtbΔ*nuoG* infected cells were analyzed using DCFDA staining. The percentage of infected cells containing one or more ROS-positive phagosomes was quantified from two donors. The amount of cells containing ROS-positive Mtb phagosomes was similar from both donors (19.1±2.9% and 21.8±1.1%). However, these percentages were increased at least 3 fold in MtbΔ*nuoG* infected cells to be 69.3±1.9% and 69.5±7.4 for the two donors (Figure 6A). Also of note, cells infected with MtbΔ*nuoG* contained many more ROS-positive phagosomes than those infected with Mtb (Figure 6B and data not shown).

### MtbΔ*katG* infection increases ROS production and induces apoptosis in macrophages

**Figure 7:**
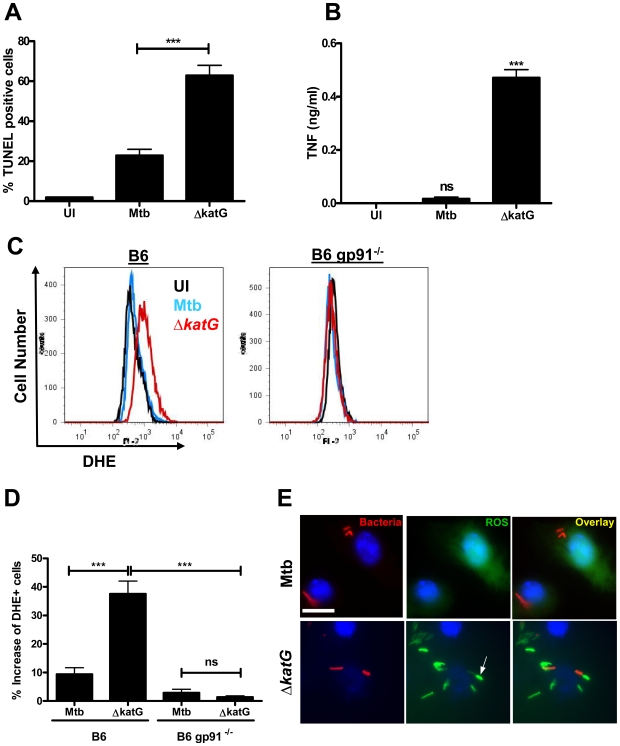
Mtb KatG mediates neutralization of phagosomal ROS to inhibit host cell apoptosis. (A) THP-1 cells were infected with wild type (Mtb) and KatG deficient Mtb (Δ*katG*) and assayed for apoptosis via TUNEL staining 3 days post infection. (B) TNF-α concentrations in culture supernatants from (A) were assayed by ELISA. (C) BMDMs from C57B/6 (B6) or gp91 deficient mice (B6 *gp91* ^−/−^) were infected with Mtb and Δ*katG*, stained with DHE at 24 hrs post infection, and quantified via flow cytometry. (D) Quantification of (C) using Overton cumulative histogram subtraction (FlowJo version 8.8.6 DMV). (E) Fluorescence microscopy of macrophages infected with DiI-labeled bacteria and stained with DCFDA (scale bar  = 10 µm). (C and E) representative samples shown. Arrows indicate phagosomal ROS accumulation.

Since the pro-apoptotic phenotype of MtbΔ*nuoG* is dependent upon the accumulation of ROS in the phagosome, we hypothesized that neutralization or countering of phagosomal ROS may be a general mechanism of inhibition of apoptosis. If this hypothesis was true, other known ROS neutralizing proteins could potentially play a role in inhibition of PCD in host cells. *M. tuberculosis* contains several enzymes involved in the neutralization of ROS including a secreted Mg, Fe superoxide dismutase (SodA), an outer membrane bound Cu, Zn superoxide dismutase (SodC), and a secreted catalase (KatG). Interestingly, a previous report established the involvement of SodA in the inhibition of apoptosis [27]. To determine if SodC or KatG could likewise affect cell death pathways, THP-1 cells were infected with *sodC* and *katG* deletion mutants and stained with TUNEL after 3 days. MtbΔ*sodC* did not induce more apoptosis than the wild type Mtb (strain Erdman) (Figure S3), possibly due to the redundant presence of secreted SodA. However, MtbΔ*katG* induced significantly more apoptosis than Mtb, both at day 3 (63±5.1% and 23±3.2%, respectively)(Figure 7A) and at day 1 (Figure S1B) post infection. Similar to cells infected with Δ*nuoG* bacteria, MtbΔ*katG* infected cells secreted 50-fold more TNF-α (0.5 ng/ml) than those infected wild type bacteria (16 pg/ml)(Figure 7B). Infection of murine macrophages with the *katG* knockout also resulted in the increase of NOX2-dependent phagosomal ROS (Figure 7C–7E). These results are consistent with the data obtained from the MtbΔ*nuoG* analysis and reinforce the hypothesis that the NOX2-mediated accumulation of phagosomal ROS can lead to induction of host cell apoptosis.

## Discussion

The search for anti-apoptosis genes in the genome of *M. tuberculosis* led to the identification of *nuoG* as being important in host cell apoptosis inhibition and bacterial virulence [26]. Here we describe that primary human alveolar macrophages and murine BMDMs infected with the *nuoG* mutant responded with a NOX2-mediated increase in phagosomal ROS, which was essential to its pro-apoptotic phenotype when compared to wild-type Mtb-infected cells. The presence of TNF-α was necessary but not sufficient for the nuoG mediated apoptosis induction. Furthermore, the infection with the nuoG mutant led to an increase in TNF-α secretion in human and murine macrophages. It is to our knowledge the first time that a direct connection between phagocytosis of a pathogen, NOX2-generated phagosomal ROS levels, and TNF-α-mediated apoptosis signaling has been demonstrated in infected macrophages.

TNF-α receptor 1 (TNF-R1) mediated signaling has either pro-survival or pro-apoptotic consequences [32]. The ligation of TNFR-1 results in either activation of NF-κB, leading to survival of the cell, or activation of the c-Jun N-terminal kinase (JNK), which entails an apoptotic response [32], [35]. A major determinant in the outcome of TNF-α-mediated cell signaling is the concentration of cytosolic ROS [36]. High ROS levels lead to oxidation and inactivation of the MAP Kinase Phosphatases (MKPs), which in their active form inhibit JNK activity. Without active MKP, TNF-α signaling leads to prolonged activation of JNK and subsequent cell death [33]. We have clearly demonstrated that intracellular ROS levels are important for the apoptosis phenotypes of the *nuoG* and *katG* deletion mutants of Mtb (Figure 3+7). It will be of interest to determine if the increased phagosomal ROS during mutant Mtb-infection leads to the oxidation of MKPs, or if other components are involved to modify TNF-α signaling outcome. How the increase in phagosomal ROS actually affects cytosolic host cell signaling components is not a trivial question. NOX2-complex generated superoxide is impermeable to lipid bilayer of the phagosome[37]. In contrast, hydrogen peroxide is highly permeable and might thus quickly diffuse into the host cell cytosol to oxidize susceptible cysteines in signaling proteins. Indeed, the JNK phosphatases MKP-1, MKP-3, MKP-5 and MKP-7 all share a phosphatase domain that contains a cysteine which is oxidized upon increase of H_2_O_2_ to inactivated the phosphatase and thus lead to increased JNK activity[33]. Nevertheless, H_2_O_2_ diffuses rapidly and so it would be surprising that we could detect such a strong accumulation of ROS in the phagosome of infected cells (Figures 4D, 6B and 7E). An alternative hypothesis is that the increase in phagosomal ROS leads to a change in the signaling of receptors in the phagosomal membrane. Cell surface receptors such as TLRs, MARCO and TNF-R1 are phagocytosed together with bacteria in macrophages and the content of the phagosome influences outcome of receptor signaling [38], [39], [40], [41]. The highly oxidative environment of the phagosome containing mutant Mtb compared to wild-type Mtb, may lead to the modification of the ligand/receptor-interactions which could affect the outcome of the signals transmitted by the receptors. The signaling difference observed between non-oxidized and oxidized LDL may serve as an example of how the oxidative modification of a ligand affects receptor signaling [42].

Infection of macrophages with either the *katG* mutant or the *nuoG* deletion mutant of Mtb increased the amount of secreted TNF-α (Figures 2A, 2B and 7B). Infection of macrophages with wild-type Mtb induces a low basal level of TNF-α secretion which is induced by transcriptional upregulation of TNF-α mRNA expression[43]. The Mtb mutant mediated increase in TNF-α secretion in human THP-1 cells was inhibited by glutathione and DPI but was not affected in murine BMDMs derived from NOX2-deficient mice (Figures 3B and 3D). This would suggest that there are functional differences between the human macrophage-like cell line THP-1 and murine BMDMs. Alternatively, the TNF- α induction might be mediated via ROS generated in mitochondria which would be inhibited by glutathione and DPI but not by deletion of NOX2. How the *nuoG* mutant infection leads to an increase in TNF-α secretion by macrophages needs to be investigated in more detail but activation of transcription factors such as ATF-2, Elk-1 and c-Jun upon JNK activation have been reported and would lead to an increase in TNF-α gene transcription[35].

The respiratory burst associated with Mtb infection has been shown to rapidly induce a MAP kinase cascade and NF-κB activation in a NOX2-dependent manner during very early time points (<1 hr post infection)[44]. However, our data suggests that ROS signaling may also play a role at later stages of infection as NOX2-derived ROS are necessary for induction of apoptosis several days post infection (Figure 3). Comparing the effects of Mtb and the *nuoG* mutant should prove to be a useful model for elucidating the interactions of NOX2-generated phagosomal ROS levels on the host cell apoptosis signaling cascade after prolonged infection.

The specific mechanism by which NuoG inhibits ROS accumulation in the phagosome remains to be determined. However, one potential mechanism could be via the direct neutralization of NOX2 generated superoxides, since they are able to oxidize iron-sulphur ([Fe-S]) clusters with extremely high efficiency[37]. The Mtb NuoG protein contains four [Fe-S] clusters which could directly compete for NOX2 generated superoxides. Nevertheless, this model would require NuoG to enter the lumen of the phagosome, and to date there is no evidence that NuoG is being secreted by Mtb. NuoG does not have a signal peptide and structural analyses of other bacterial NDH-1 systems predict NuoG to be in the cytosol of the bacteria [45]. Furthermore, we have previously failed to detect secretion of a NuoG-phoA fusion protein[26] and in the current manuscript we also did not observe secretion of a myc-tagged NuoG protein into culture filtrate (Figure S4). These results are significant as they suggest that it is not NuoG by itself that is important for phagosomal superoxide neutralization, but that it is potentially the enzymatic activity of the NDH-1 complex that it is required. In order to address this question experimentally, deletion mutants of the NuoL and NuoM subunits of NDH-1 will be created. In homology with other prokaryotic NDH-1 complexes the L and M subunits are proposed to be important in translocation of protons across the membrane during the dehydrogenase activity of NDH-1 and thus their deletion should abolish the enzymatic activity of the NDH-1 complex[45]. If the hypothesis that the enzymatic activity of the NDH-1 complex is important for NOX2 neutralization is valid, then these deletion mutants should have a similar phenotype to the *nuoG* mutant in regard to ROS and apoptosis increases in host macrophages.

In the light of our results it is tempting to hypothesize that the NDH-1-encoding *nuo*-operon in *M. tuberculosis* might have acquired a different function when compared to other prokaryotes. Accordingly, regulation of the Mtb *nuo*-operon is opposite to that in *E. coli*. In Mtb, gene expression of the nuo-operon is down-regulated under hypoxic conditions *in vitro* and at late stage infections in the lungs of mice, whereas it is upregulated under these conditions in *E.coli* [46]. Interestingly, it is the type II dehydrogenase complex, NDH-2 (*ndh*, *ndhA*), of Mtb that is upregulated under hypoxic, nonreplicating conditions[46]. Under these conditions NDH-2 is crucial for maintaining a minimal PMF which is essential for survival[47], suggesting a possible alternative role for the Mtb NDH-1 system. The *nuo*-operon is under positive control by the two-component system PhoPR [48], which is important for virulence of Mtb and is one of the targets for attenuating mutations in Mtb H37Ra[49], [50]. The *phoP* mutant fails to replicate in macrophages and infected mouse organs; however bacteria are able to survive in a state of nonreplicating persistence, suggesting that the dormancy regulon is not affected by the *phoP* mutation and that the PhoPR system is important for early steps of Mtb infection[51]. This is consistent with a role of the NDH-1 complex during the replicative phase of Mtb infections when the host cell NOX2 system is the most active.

The NOX2 complex has been investigated and demonstrated to be of great importance for innate immune defense against a variety of pathogens[5]. In order for bacterial or protozoal pathogens to survive inside the macrophage they must have developed strategies to overcome NOX2 activity. One approach is to directly inhibit NOX2 activity by either perturbing the recruitment of the subunits to the phagosome[3], [52] or by decreasing the steady state levels of NOX2 complex subunits[7], [8]. A novel mechanism employed by *Helicobacter pylori* is to misdirect the assembly of functional NOX2 complex away from the membrane of phagosome to the plasma membrane, so that superoxides are being released into the extracellular space instead of the phagosomal lumen[6]. Furthermore, a common strategy used by several pathogenic bacteria, including *M. tuberculosis,* is the enzymatic detoxification of NOX2 generated superoxides via the secretion of enzymes such as superoxide dismutases and catalases[53]. In the case of Mtb, the secretion of large amounts of SodA and KatG may account for the relative insensitivity of the bacteria to bactericidal effects of NOX2 produced superoxides[54]. If our discovery that the NuoG-mediated neutralization of NOX2 activity is important for inhibition of host cell apoptosis is of general importance, one would predict that any mutant deficient in inhibition NOX2 activity should have a pro-apoptotic phenotype. There are few defined mutants for any pathogen described that are deficient in neutralizing NOX2 activity, and could thus be used to confirm or disprove this hypothesis. In the present study, we interrogated a Mtb deletion mutant of the only catalase in the Mtb genome (*katG*) and demonstrated that it had a similar phenotype to the *nuoG* mutant of Mtb in regard to an increase in phagosomal ROS and host cell apoptosis induction, both of which were dependent upon functional NOX2 (Figure 7). Interestingly, the *katG* mutant has been described as being attenuated and the attenuation was dependent on the presence of functional NOX2 complex in the host[55]. Furthermore, inhibition of SodA secretion by Mtb achieved either via deletion of *secA2* or via inhibiting *sodA* transcription also leads to a pro-apoptotic phenotype of the bacteria[27]. This increase in apoptosis is likely to be due to increases in phagosomal ROS levels and dependent upon host cell NOX2 activity, although that has not been investigated to date.

*Mycobacterium tuberculosis* also contains the membrane bound superoxide dismutase SodC. We have found that the deletion of *sodC* does not result in a pro-apoptotic phenotype, likely due to the presence of secreted SodA (Figure S3). However, this deletion does render the mutant more susceptible to the bacteriacidal effects of ROS [56]. It is possible that the deletion of *nuoG* from Mtb may also create a bacterial strain that is more vulnerable to ROS mediated killing, in which case the pro-apoptotic phenotype of Δ*nuoG* may be due to decreased fitness of the mutant. However, the *nuoG* deletion mutant was not more susceptible to superoxides being added directly to bacteria using the hypoxanthine/xanthine oxidase system (Figure S5). Therefore, the increase in phagosomal ROS may be affecting apoptosis signaling rather than direct bacterial killing.

The identification of both SodA and KatG as anti-apoptotic proteins indicate that for Mtb, mutants deficient in countering host cell NOX2 activity are generally pro-apoptotic. It will be interesting to know if this mechanism can be extended to other pathogens such as *Leishmania donovani*, which is able to inhibit host cell NOX2 recruitment to the phagosome. This hypothesis is testable as a mutant deficient in producing the surface glycolipid lipophosphoglycan has lost the capacity to inhibit NOX2 recruitment [52].

Other pathogens, such as *Listeria monocytogenes*, may evade NOX2 activity by escaping from the phagosome into the cytosol. This is clearly a successful approach to evading the detrimental effects of increased proteolytic activity associated with phagosome maturation. Nevertheless, in the light of our results it is tempting to speculate that this strategy also helps to evade the NOX2-mediated apoptosis induction. It will interesting to test this hypothesis using bacterial mutants that fail to escape the phagosome such as the Listeriolysin O mutant of *Listeria monocytogenes*.

In conclusion, the investigation of the pro-apoptotic phenotype of a mutant of Mtb deficient in functional NDH-1 complex serendipitously revealed a novel important function of host cell NOX2 complex in macrophages. Our results demonstrate that continuous NOX2 activity will ultimately lead to host macrophage apoptosis induction. The classical respiratory burst is transient, since this generates sufficient amounts ROS to kill susceptible bacteria and thus reduce NOX2 activity. However, infection of macrophages with persistent pathogens, who have adapted to the macrophage as a survival niche and are able to survive this initial ROS burst, would thus potentially lead to continuous NOX2 activity. The results presented in the current manuscript enable us to formulate the following hypothesis: successful intracellular pathogens need strategies to inhibit prolonged activation of NOX2 and/or neutralize the generated superoxides since this will otherwise be sensed by the host cell and will lead to host cell apoptosis. This hypothesis expands the function of NOX2 from the previously described ROS generation for bactericidal activity, to postulate that the host cell macrophages use the NOX2 complex as a mechanism to detect persisting intracellular pathogens.

## Materials and Methods

### Ethics statement

This study was conducted according to the principles expressed in the Declaration of Helsinki. The study was approved by the Institutional Review Board of the University of Maryland. All patients provided written informed consent for the collection of samples and subsequent analysis.

All animals were handled in strict accordance with protocols approved by the Institutional Animal Care & Use Committee of the University of Maryland (protocol #R-09-35).

### Materials

C57/B6 and GP91 knockout mice were obtained from Jackson laboratories (www.jaxmice.jax.org). Caspase specific inhibitors and analogs were purchased from Calbiochem (www.emdbiosciences.com). Neutralizing anti human-TNF antibody (#500-M26), the biotinylated detection antibody (500-P31Abt) were purchased from Peprotech Inc (www.peprotech.com). Recombinant human and murine TNF-α, and anti murine-TNF-α antibodies were purchased from BD Pharmingen (www.bdbiosciences.com). CM-DCFDA, DHE, and Vybrant® DiI cell-labeling solution were purchased from Invitrogen (www.invitrogen.com). All other reagents unless otherwise noted were purchased from Sigma (www.sigma.com).

### Bacteria and culture conditions

*M. tuberculosis* H37RV (ATCC 25618) was obtained from the American Type Culture Collection (www.atcc.org), MtbΔ*katG* was obtained from TARGET (http://webhost.nts.jhu.edu/target/Default.aspx), and MtbΔ*nuoG* has been previously described [26]. GFP expressing Mtb and Δ*nuoG* were created by transfecting the GFP-pmV261 plasmid into competent cells by electroporation as previously described [26]. All mycobacteria, excluding Δ*katG*, were grown in 7H9 media supplemented with 0.5%glycerol, 0.5% Tween-80, and 10% ADS. Δ*KatG* was grown in the same media supplemented with ADC in place of ADS. For selective media, 50 µg/ml Hygromycin or 25 µg/ml Kanamycin were added.

### Cell culture conditions and infection

Human myelomonocytic cell line THP1 (ATCC TIB-202) was cultured in RPMI (ATCC) supplemented with 10% heat inactivated FCS (Hyclone) and differentiated using 20 ng/ml phorbol myristate acetate (PMA)(Sigma) as described [26]. Bacteria were grown to an OD_600_ ranging from 0.5 to 0.8 and the culture was allowed to settle for 10 minutes. Infections were carried out at a multiplicity of infection (MOI) of 5∶1 (5 bacilli to 1 cell) for 4 hours in infection media containing 10% human serum (Sigma) and 10% non heat inactivated FCS. After 4 hours, extracellular bacteria were removed by 2 washes with phosphate buffered saline (PBS) and the cells were incubated in chase media containing 100 µg/ml of gentamicin (Invitrogen). Cells were assayed for apoptosis by TUNEL staining 3 or 5 days post infection as detailed in the figure legends. The protocol to obtain normal human bronchoalveolar lavage fluid (BALF) was pre-approved by the IRB of the University of Maryland-Baltimore (H-23204). Normal, asymptomatic, non-smoking volunteers between the ages of 18 and 50 were anesthetized with topical and endobronchial lidocaine, and clinically standard fiberoptic bronchoscopy (FOB) was performed in the endoscopy suite at the University of Maryland Hospital. BALF was obtained using 200 mL of sterile normal saline infused in an identical manner into the right middle lobe of each subject, yielding 75–125 mL of BALF. 10–15 mL of BALF was filtered through sterile gauze to remove mucous, and the alveolar macrophages were washed 3 times with PBS before being used in experiments. Cells were resuspended in warm RPMI with 10% heat inactivated FCS, seeded on 8 well slides, and allowed to rest for 1–3 days. Infection was carried out as described above. Bone marrow macrophages were derived from the femur and tibia of C57B/6 and knockout mice and differentiated in DMEM media containing 20% L-929 supernatant. Murine cells were infected as described above using 10% FCS and 5–10% L929 supernatant in the infection and chase media. L929 supernatant was included in order to protect against cytokine withdrawal induced apoptosis. For experiments using caspase inhibitors or analog (20 µM), antioxidants (15 mM glutathione), and oxidase inhibitor (10 µM diphenylene iodonium, DPI), the cells were incubated with the reagents during infection and chase period. In experiments using TNF-α neutralizing antibody (#500-M26, Peprotech) the antibody was included only in the chase medium at a concentration of 5 µg/ml.

### Apoptosis assays

The TUNEL assay was preformed to reveal apoptosis-induced DNA fragmentation in tissue culture, primary human, or murine cells using the “In Situ Cell Death Detection Kit-Fluorescein or –TMR Red” (Roche Applied Sciences at roche.com). The assay was carried out as described by the manufacturer and the percentage of stained cells was analyzed using flow cytometry or quantification via fluorescent microscopy.

### ROS detection assays

Reactive oxygen species in primary murine bone marrow cells and alveolar macrophages were detected at 24 hrs or 3 days post infection respectively using the ROS sensitive dyes 5-(and-6)-chloromethyl-2′,7′-dichlorodihydrofluorescein diacetate, acetyl ester (CM-DCFDA) and dihydroethidium (DHE) (Invitrogen). Bone marrow cells were deprived of L929 supernatant 16 hrs prior to infection and maintained in L929 free media without phenol red for the length of the experiment. Human alveolar macrophages were maintained in normal growth, infection, and chase media. In some cases bacteria were labeled with lipophilic red dye Vybrant-DiI (invitrogen). Bacteria were incubated in 7H9 media containing 5 µl/ml of DiI for 30 minutes, washed twice with PBS with 0.05%tween, and then used for infection as normal. Post infection, murine or alveolar macrophages were washed once in HBSS and then incubated in 10 µM DCFDA for 30 minutes or 2 µM DHE for 15 minutes. Cells were washed 3 times with HBSS, fixed with 4% paraformaldehyde, and analyzed by either flow cytometry or fluorescence microscopy.

### Nitrite detection assay

Nitrite (NO) concentrations in supernatants from C57/B6 BMDMs were quantified via the Griess assay according to the manufacturer's protocol. In brief, supernatants were collected from macrophagess 3 days post infection with Mtb or Δ*nuoG*. Supernatants from macrophages primed for 16 hrs with IFNγ (100 units/ml), and infected with heat-killed *M. smegmatis* (MOI of 5 and 20), were used as positive controls. 150 µl of sample was mixed with 20 µl of Greiss reagent and 130 µl water, and the mixture was incubated at 30°C for 30 minutes before measuring absorbance at 548 nm. Nitrite concentrations were calculated from a standard curve with sodium nitrite as the reference.

### TNF-α ELISA

ELISA was performed with the supernatants of bone marrow derived macrophages or THP-1 cells infected for 3 or 5 days and treated with or without glutathione or DPI as described above. For detection of human TNF-α, the ELISA-plates were coated with 2 µg/ml capture antibody (500-M26, Peprotech) for 2 hours at 37°C. 100 µl of cell supernatant was used for the reaction and recombinant human TNF-α (554618, BD Pharmingen) diluted in infection medium was used as a standard. TNF-α was detected using the secondary biotinylated anti human-TNF-α detection antibody at 200 ng/ml (500-P31Abt, Peprotech Inc), Streptavidin-alkaline phosphatase at 1 µg/ml (Zymed), and phosphatase substrate at 1 mg/ml (Sigma). The plate was read at an absorbance of 405 nm. Murine TNF-α ELISAs were preformed as above using recombinant mouse TNF-α standard, the capture antibody rat anti murine-TNF-α at 8 µg/ml, and the biotinylated detection antibody rat anti mouse-TNF-α antibody at 1 µg/ml (Catalog numbers 554589, 551225, 554415 respectively, BD Pharmingen).

### Statistical analysis

Statistical analyses were performed on three independent experiments (ANOVA with Tukey post-test) unless otherwise noted in the figure legends. Significance indications are as follows: *, 0.01<p<0.05; **, 0.001<p<0.01; ***, p<0.001. Graphs and in –text citations are a representation of the population mean and standard error of mean. Percentages of DCFDA or DHE positive cells found in the sample and not the control (Figure 4 and Figure 7) were calculated by subtracting the histogram of uninfected cells from experimental histograms using Overton cumulative histogram subtraction (FlowJo version 8.8.6 DMV). Differences were compared via ANOVA.

## Supporting Information

Figure S1 The pro-apoptotic phenotypes of the (A) *nuoG* and (B) *katG* deletion mutants are observed at the same time points as increased ROS is detected. B6 BMDMs were starved from cytokines (L929 supernatant) for 16hrs prior to infection with Δ*nuoG* or Δ*katG* as according to the ROS detection protocol. Apoptotic cells were quantified 24 hpi via TUNEL staining.
(0.53 MB TIF)

Figure S2 Nitric oxide production by infected THP-1 cells. Concentrations of NO in supernatants from uninfected (UI), IFNγ and LPS treated, Mtb or the *nuoG* deletion mutant (Δ*nuoG*) infected THP-1 cells were assayed via the Griess assay. Supernatants from IFNγ and LPS treated cells were collected after 18 hrs, whereas supernatants from infected and uninfected cells were assayed 3 days post infection.
(0.52 MB TIF)

Figure S3 The *sodC* deletion mutant does not have a pro-apoptotic phenotype. THP-1 cells were infected with either Mtb-Erdman or the *sodC* deletion mutant at an MOI of 10 for 4 h and assayed for apoptosis after 3 days by TUNEL staining (Mean+/− SEM of three experiments).
(0.52 MB TIF)

Figure S4 Mtb NuoG is not secreted into the culture filtrate. *NuoG* knockout bacteria were complemented with a NuoG-myc construct and grown in Sauton's media to an OD_600 nm_ of 0.7. Shown is a western blot of NuoG-Myc (92KDa) on culture filtrate (CF) and the bacterial pellet (P). Equal ratios of protein were loaded for CF and P. Antibodies against Antigen 85 (32KDa) were used as a loading control and to show that the CF contained protein (representative sample shown).
(0.66 MB TIF)

Figure S5 The *nuoG* deletion mutant has no increased sensitivity to superoxide-dependent killing. The resistance of the *nuoG* mutant (open squares) to ROS was compared to survival of wild-type Mtb (open circles) using hypoxanthine/xanthine oxidase to generate 0_2_^-^ in the bacterial culture medium. The number of surviving bacteria was determined at 0, 1, and 3 h after exposure to superoxide *in vitro* by plating dilutions of the bacteria on 7H10 plates. Viability of Mtb (closed circles) and Δ*nuoG* (closed squares) not exposed to xanthine oxidase were also determined. The means from triplicate tubes were calculated, and the data are expressed as the mean percentages of the time zero value with SEM.
(0.52 MB TIF)
