# Supplementary figures and images for: The Type I NADH Dehydrogenase of Mycobacterium tuberculosis Counters Phagosomal NOX2 Activity to Inhibit TNF-α-Mediated Host Cell Apoptosis
Source: PLoS Pathog. 2010 Apr 22;6(4):e1000864. doi: 10.1371/journal.ppat.1000864 (PMC2858756; doi:10.1371/journal.ppat.1000864)

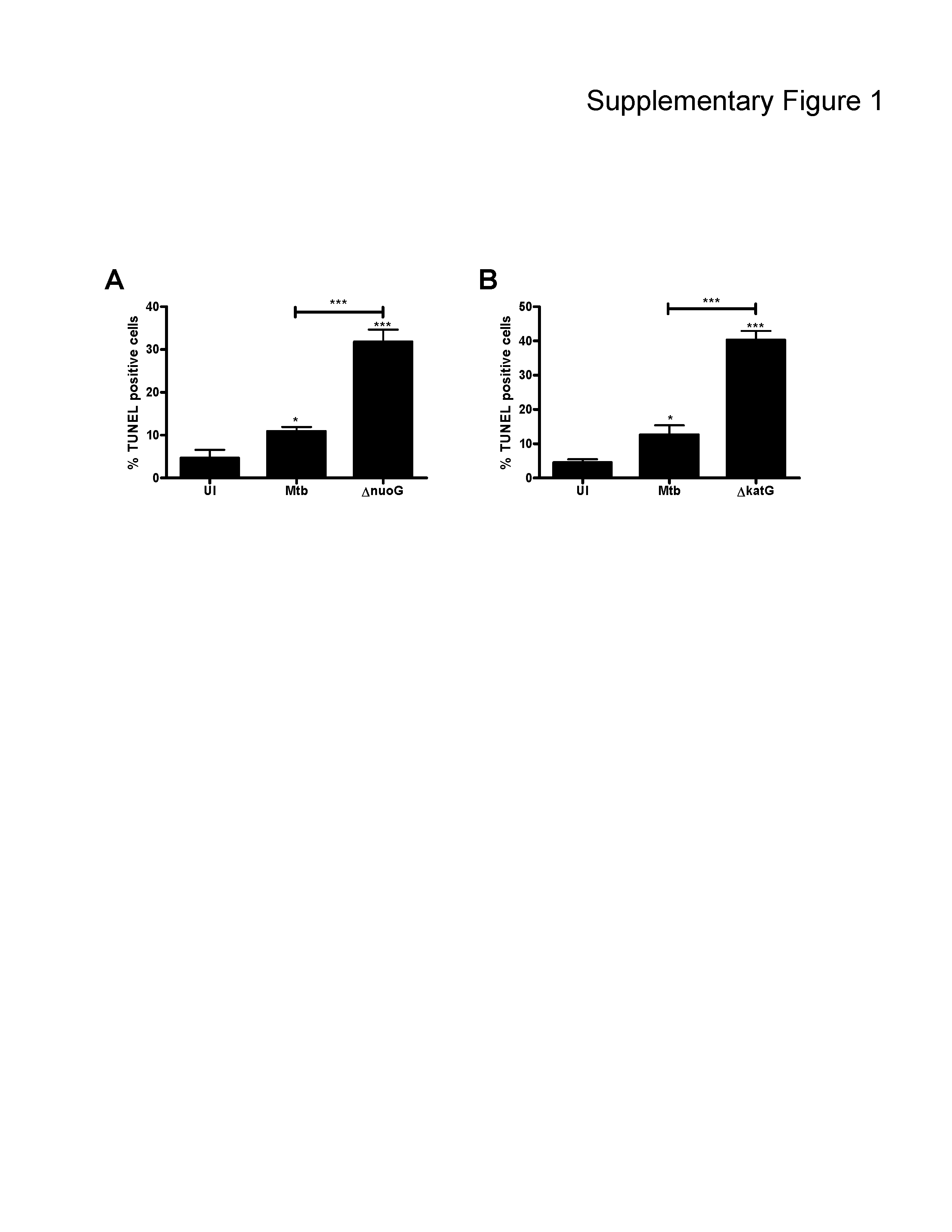

Supplement: Figure S1 — The pro-apoptotic phenotypes of the (A) nuoG and (B) katG deletion mutants are observed at the same time points as increased ROS is detected. B6 BMDMs were starved from cytokines (L929 supernatant) for 16hrs prior to infection with ΔnuoG or ΔkatG as according to the ROS detection protocol. Apoptotic cells were quantified 24 hpi via TUNEL staining. (0.53 MB TIF) [file ppat.1000864.s001.tif]

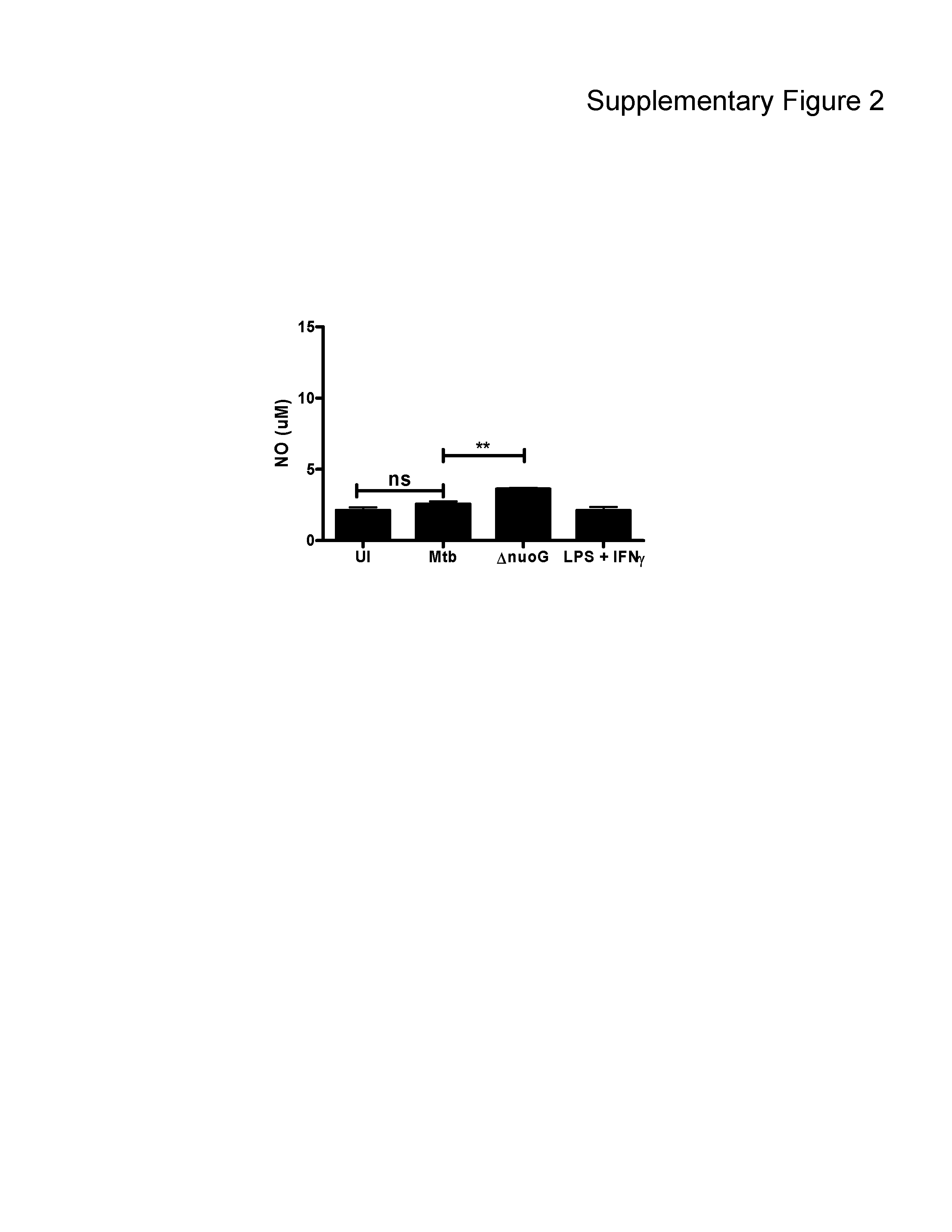

Supplement: Figure S2 — Nitric oxide production by infected THP-1 cells. Concentrations of NO in supernatants from uninfected (UI), IFNγ and LPS treated, Mtb or the nuoG deletion mutant (ΔnuoG) infected THP-1 cells were assayed via the Griess assay. Supernatants from IFNγ and LPS treated cells were collected after 18 hrs, whereas supernatants from infected and uninfected cells were assayed 3 days post infection. (0.52 MB TIF) [file ppat.1000864.s002.tif]

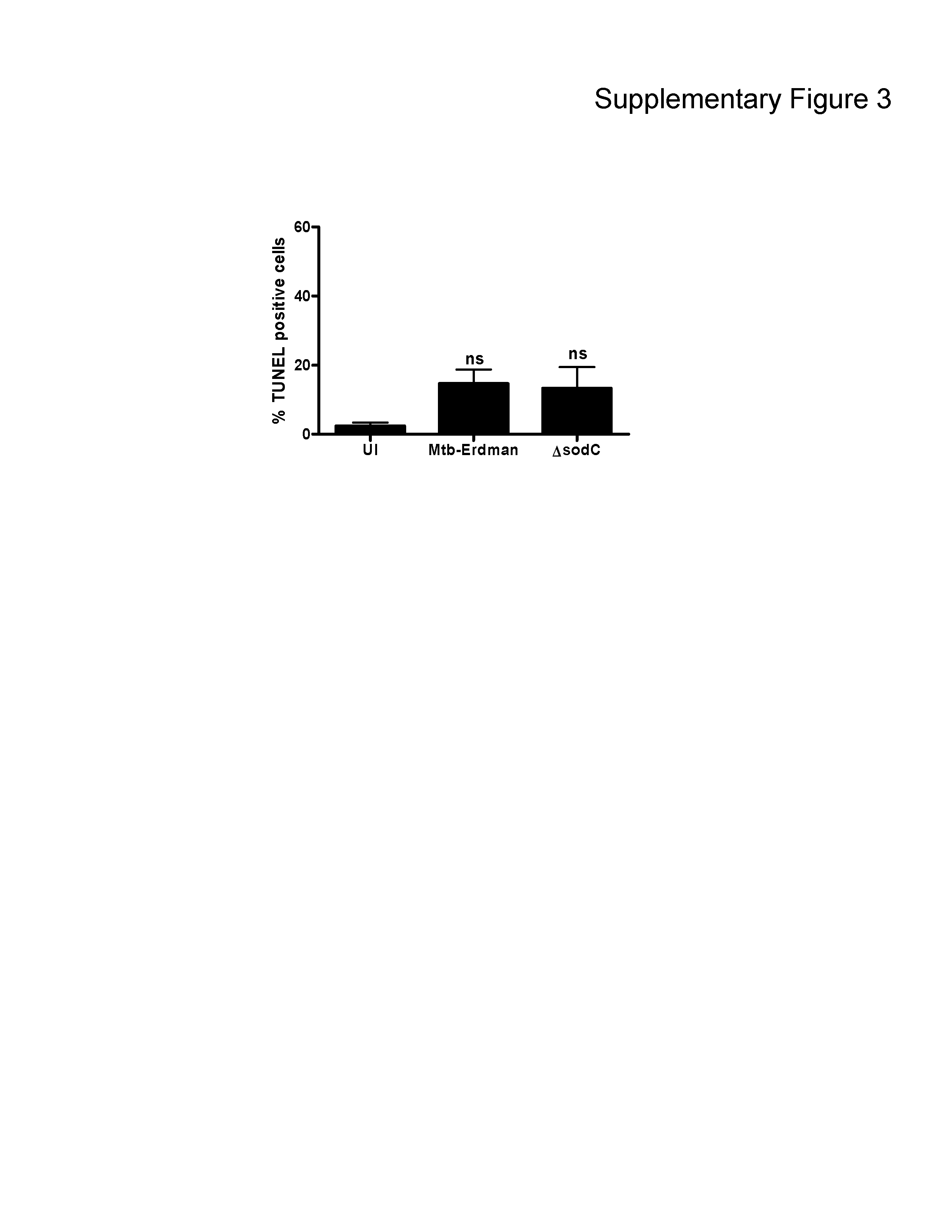

Supplement: Figure S3 — The sodC deletion mutant does not have a pro-apoptotic phenotype. THP-1 cells were infected with either Mtb-Erdman or the sodC deletion mutant at an MOI of 10 for 4 h and assayed for apoptosis after 3 days by TUNEL staining (Mean+/− SEM of three experiments). (0.52 MB TIF) [file ppat.1000864.s003.tif]

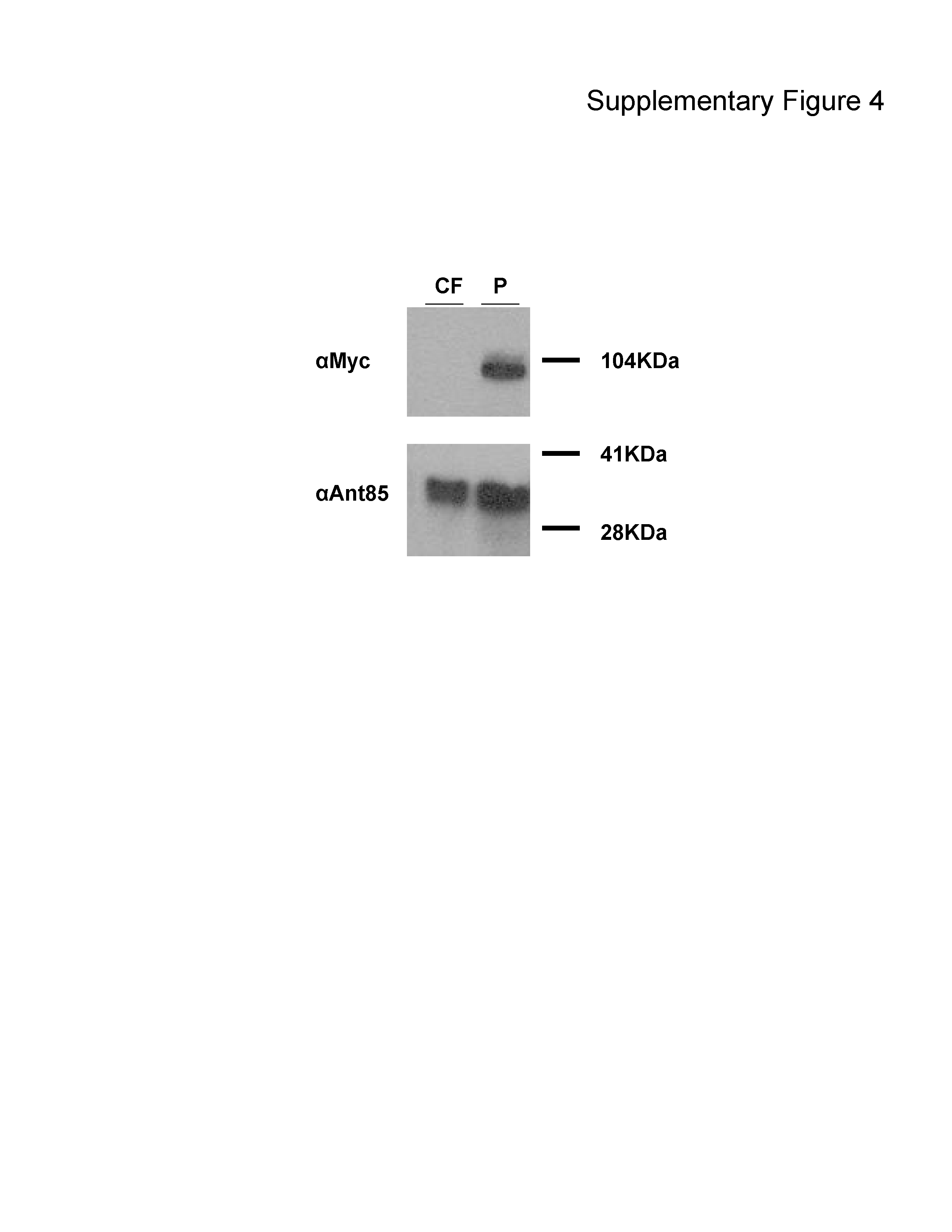

Supplement: Figure S4 — Mtb NuoG is not secreted into the culture filtrate. NuoG knockout bacteria were complemented with a NuoG-myc construct and grown in Sauton's media to an OD600 nm of 0.7. Shown is a western blot of NuoG-Myc (92KDa) on culture filtrate (CF) and the bacterial pellet (P). Equal ratios of protein were loaded for CF and P. Antibodies against Antigen 85 (32KDa) were used as a loading control and to show that the CF contained protein (representative sample shown). (0.66 MB TIF) [file ppat.1000864.s004.tif]

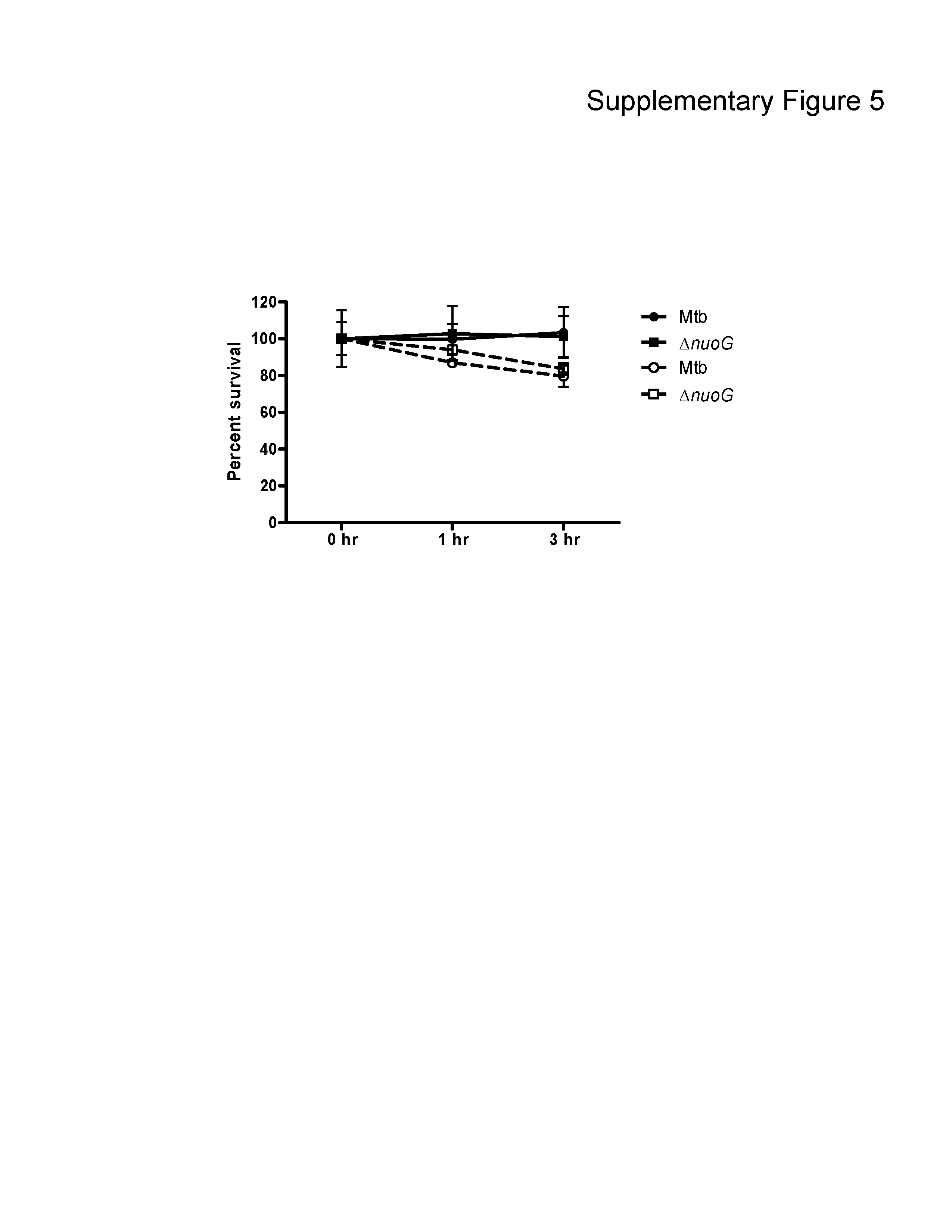

Supplement: Figure S5 — The nuoG deletion mutant has no increased sensitivity to superoxide-dependent killing. The resistance of the nuoG mutant (open squares) to ROS was compared to survival of wild-type Mtb (open circles) using hypoxanthine/xanthine oxidase to generate 02 - in the bacterial culture medium. The number of surviving bacteria was determined at 0, 1, and 3 h after exposure to superoxide in vitro by plating dilutions of the bacteria on 7H10 plates. Viability of Mtb (closed circles) and ΔnuoG (closed squares) not exposed to xanthine oxidase were also determined. The means from triplicate tubes were calculated, and the data are expressed as the mean percentages of the time zero value with SEM. (0.52 MB TIF) [file ppat.1000864.s005.tif]
